# The non-protein coding breast cancer susceptibility locus *Mcs5a *acts in a non-mammary cell-autonomous fashion through the immune system and modulates T-cell homeostasis and functions

**DOI:** 10.1186/bcr2933

**Published:** 2011-08-16

**Authors:** Bart MG Smits, Deepak Sharma, David J Samuelson, Stephan Woditschka, Bob Mau, Jill D Haag, Michael N Gould

**Affiliations:** 1 McArdle Laboratory for Cancer Research, Department of Oncology, University of Wisconsin School of Medicine and Public Health, University of Wisconsin-Madison, 1400 University Avenue, Madison, WI 53706, USA; 2 Department of Biochemistry and Molecular Biology, University of Louisville School of Medicine, 319 Abraham Flexner Way, Louisville, KY 40292, USA; 3 National Cancer Institute, Cancer Prevention Fellowship Program, 6120 Executive Boulevard, Bethesda, MD 20892, USA

## Abstract

**Introduction:**

Mechanisms underlying low-penetrance, common, non-protein coding variants in breast cancer risk loci are largely undefined. We showed previously that the non-protein coding mammary carcinoma susceptibility locus *Mcs5a/MCS5A *modulates breast cancer risk in rats and women. The *Mcs5a *allele from the Wistar-Kyoto (WKy) rat strain consists of two genetically interacting elements that have to be present on the same chromosome to confer mammary carcinoma resistance. We also found that the two interacting elements of the resistant allele are required for the downregulation of transcript levels of the *Fbxo10 *gene specifically in T-cells. Here we describe mechanisms through which *Mcs5a *may reduce mammary carcinoma susceptibility.

**Methods:**

We performed mammary carcinoma multiplicity studies with three mammary carcinoma-inducing treatments, namely 7,12-dimethylbenz(a)anthracene (DMBA) and *N*-nitroso-*N*-methylurea (NMU) carcinogenesis, and mammary ductal infusion of retrovirus expressing the activated *HER2/neu *oncogene. We used mammary gland and bone marrow transplantation assays to assess the target tissue of *Mcs5a *activity. We used immunophenotyping assays on well-defined congenic rat lines carrying susceptible and resistant *Mcs5a *alleles to identify changes in T-cell homeostasis and function associated with resistance.

**Results:**

We show that *Mcs5a *acts beyond the initial step of mammary epithelial cell transformation, during early cancer progression. We show that *Mcs5a *controls susceptibility in a non-mammary cell-autonomous manner through the immune system. The resistant *Mcs5a *allele was found to be associated with an overabundance of gd T-cell receptor (TCR)+ T-cells as well as a CD62L (L-selectin)-high population of all T-cell classes. In contrast to in mammary carcinoma, gdTCR+ T-cells are the predominant T-cell type in the mammary gland and were found to be overabundant in the mammary epithelium of *Mcs5a *resistant congenic rats. Most of them simultaneously expressed the CD4, CD8, and CD161α markers. In cultured T-cells of *Mcs5a *resistant congenic rats we found increased mitogen-induced proliferation and production of Th1 cytokines IFNg, IL-2, and Tumor Necrosis Factor (TNF), but not Th2 cytokines IL-4 and IL-6, or Th17 cytokine IL-17 when compared with susceptible control rats.

**Conclusions:**

These data support a hypothesis that *Mcs5a *displays a non-mammary cell-autonomous mechanism of action to modulate breast cancer risk through the immune system. The resistant *Mcs5a *allele is associated with alterations in T-cell homeostasis and functions, and overabundance of γδTCR+ T-cells in carcinogen-exposed mammary epithelium.

## Introduction

The genetic component of risk for most common forms of breast cancer defines it as a complex trait consisting of numerous susceptibility alleles and interactions. Thus far, approximately 25 such alleles have been identified by using genome-wide association studies and comparative genetics [1-10]. A great majority of these alleles are non-protein-coding and each is associated with a low relative risk. Currently, a major open question regarding breast cancer susceptibility alleles is defining their function and risk-controlling mechanisms. Such insight will allow the use of discovered alleles to go beyond prognosis and toward the development of novel anticancer strategies and agents.

We identified the mammary carcinoma susceptibility-5a (*Mcs5a*) locus by using a comparative genetics approach and have shown that it controls breast cancer risk in both rats and women [5]. The *Mcs5 *quantitative trait locus was initially identified by linkage analysis in the backcross progeny of the mammary carcinoma susceptible Wistar-Furth (WF) rat strain and the resistant Wistar-Kyoto (WKy) rat strain [11]. Genetic fine-mapping using congenic recombinant rat lines resulted in the identification of three mammary carcinoma risk loci, one of which was *Mcs5a *[12,13]. The resistant allele of *Mcs5a*, when introgressed into the susceptible genetic background, is associated with an approximately 50% reduction in mammary carcinoma multiplicity. *Mcs5a *consists of two non-protein-coding synthetically interacting elements (*Mcs5a1 *and *Mcs5a2*) that must be located on the same chromosome to elicit the resistance phenotype. Previously, we showed that the expression levels of genes located within 1 Mb surrounding this locus, including *Fbxo10*, *Frmpd1*, and *Tomm5 *(partially) overlapping with the locus, were not differentially expressed in the mammary gland of susceptible congenic control and *Mcs5a*-resistant congenic rats. We showed that the non-protein-coding synthetically interacting elements of the resistant allele are required for the downregulation of transcript levels of the E3 ubiquitin ligase gene *Fbxo10 *in the thymus. The differential *Fbxo10 *expression between susceptible congenic control and *Mcs5a*-resistant congenic animals is detectable in immature, naïve, and activated T cells and not in ovary, brain [5], or other cells of the immune system [14]. The transcript levels of *Tomm5 *and *Frmpd1 *in immune tissues are not associated with the presence of the interacting genetic elements of the resistant allele [14]. The genetic interaction is facilitated by a human-rat conserved higher-order chromatin-folding structure [14]. In a case-control association study, a non-protein-coding breast cancer risk-associated allele was identified in each orthologous human locus (*MCS5A1 *and *MCS5A2*) to resolutions of approximately 5.7 Kb and approximately 26.1 Kb, respectively [5]. We recently found that the variants associated with lower breast cancer risk in the human orthologous loci *MCS5A1 *and *MCS5A2 *are located at both sides of the looped structure and functionally interact to downregulate transcriptional activity of reporter constructs transfected into a human T-lymphocytic cell line [14]. *MCS5A2 *(marked by the single-nucleotide polymorphism rs2182317) was recently verified as a breast cancer risk allele, modifying risk to both estrogen receptor-positive and -negative breast cancers [4].

We hypothesize that similar mechanisms underlie the rat and human *Mcs5a/MCS5A *breast cancer susceptibility alleles given that these reside in orthologous genomic intervals, show similar higher-order chromatin structure, and control highly similar complex traits. Congenic rat lines provide a unique opportunity to investigate the activities of this locus on the level of specific cell types in a mammalian model organism. *Mcs5a *is shown here to act on mammary carcinoma multiplicity beyond the initial stage of mammary epithelial cell transformation, in a non-mammary cell-autonomous manner through the immune system, and to alter homeostasis and function of specific T-cell populations.

## Materials and methods

### Animals

The congenic rat lines were established and maintained in a facility approved by the Association for Assessment and Accreditation of Laboratory Animal Care, as previously published [13]. All animal protocols were approved by the University of Wisconsin Medical School Animal Care and Use Committee. Congenics are defined as genetic lines that were developed on a WF (susceptible) genetic background and that carry the selected WKy (resistant) *Mcs5a *alleles. Resistant congenic lines ('*Mcs5a*'; lines WW and O) with decreased susceptibility phenotypes are WKy-homozygous at the entire *Mcs5a *locus [5]. The susceptible congenic control line ('susc.'; line WF.WKy) derived from the O congenic line is WF-homozygous at *Mcs5a *and all other identified *Mcs *loci. Other susceptible congenic lines are WKy-homozygous at *Mcs5a1 *('*Mcs5a1*'; line B3) and WKy-homozygous at *Mcs5a2 *('*Mcs5a2*'; line LL) [5].

### Carcinogenesis

Female rats (age of 50 to 55 days) were orally gavaged with 7,12-dimethylbenz(a)anthracene (DMBA) at 65 mg/kg of body weight or were injected intraperitonially with *N*-nitroso-*N*-methylurea (NMU) at 50 mg/kg of body weight or were subjected to mammary ductal infusion of replication-defective retrovirus expressing the activated *HER2/neu *oncogene (*HER2/neu*) at a concentration of 5 × 10^5 ^to 1 × 10^6 ^colony-forming units (CFU) per milliliter [15]. To obtain *in situ *carcinomas (ISCs), female rats of 50 to 55 days of age were subjected to *HER2/neu *infusion at 1 × 10^7 ^CFU/mL [16]. To obtain multiplicities, mammary carcinomas of greater than 3 × 3 mm were counted at 15, 17, and 8 weeks after DMBA, NMU, or *HER2/neu *treatment, respectively, and ISCs were determined by counting individual carcinomas in whole-mounted abdominal mammary glands stained with aluminum carmine at 16 days after treatment. Multiplicity data were statistically analyzed by using Mann-Whitney non-parametric tests.

### Mammary gland transplantation

Donor mammary glands with lymph nodes (LNs) excised (both abdominal and adjacent inguinal glands) from 30- to 35-day-old females were finely minced over ice and divided into four equal volumes. One volume was transplanted onto the interscapular white fat pad of each 30- to 35-day-old recipient (one donor per four recipients). Three weeks after transplantation, all recipients were treated with DMBA as described above. At 15 weeks after DMBA, interscapular fat pads were examined for carcinoma development. In addition, each fat pad was whole-mounted and stained with aluminum carmine to verify mammary gland establishment. As only 11 out of 213 rats developed multiple carcinomas in the transplant sites, the data were analyzed as a binary response by logistic regression. The four transplant groups (donor to recipient: susceptible to susceptible, S:S; susceptible to resistant, S:R; resistant to susceptible, R:S; and resistant to resistant, R:R) form a 2 × 2 factorial design with donor and recipient genotypes as the main effects. Standard logistic regression was applied to the binary response data with two main effects and an interaction term.

### Bone marrow transplantation

At 30 to 35 days of age, recipient rats were irradiated twice with 400 (2 × 400) rads each time. The second radiation dose was given 3 hours after the first dose. Within 6 hours after irradiation, recipients were given 1 × 10^6 ^bone marrow cells from 30- to 35-day-old donors via tail vein injection. Recipients were treated with DMBA 4 weeks after transplantation. At 15 weeks after DMBA, mammary carcinomas of at least 3 × 3 mm were counted. Transplanted donor alleles were quantified by using TaqMan allelic discrimination on recipient whole-spleen genomic DNA. The quantities of transplanted donor alleles were estimated from a standard curve approach that was anchored by incorporated dilutions of WF- and WKy-homozygous DNA. The TaqMan probes (Applied Biosytems, Foster City, CA, USA) were designed to genotype a single-nucleotide polymorphism at chr*5*:61,634,727 in v3.4 of the rat genome.

### Flow cytometry

In all flow cytometry experiments, cells were stained with fluorophore-conjugated antibodies against rat CD3, CD4, CD8, CD62L, or γδTCR (BD Biosciences, San Jose, CA, USA) or isotype controls in serum-containing media or phosphate-buffered saline/Hepes. For T-cell phenotyping, spleen or mammary inguinal LN cells were obtained by squeezing the tissue through a sterile mesh in a Petri dish containing RPMI medium. The red blood cells were lysed by a brief hypotonic shock. To obtain mammary ductal fragments, mammary glands with LN excised (both abdominal and the adjacent inguinal glands) from DMBA-treated (4 weeks after treatment) and age-matched untreated females were finely minced over ice. Each sample was exposed to 10 mL of GIBCO Dulbecco's modified Eagle's medium/F12 (DMEM/F12) (Invitrogen Corporation, Carlsbad, CA, USA) containing 0.01 g/mL of type III collagenase (Worthington Biochemical Corporation, Lakewood, NJ, USA) for 2 hours at 37°C under gentle rotation. DNAseI (Worthington Biochemical Corporation) was added to 0.2 μg/mL, and the samples were incubated for 10 minutes under vigorous shaking. Fat was removed from the pelleted cell fraction by pipetting, and the pellet was washed once with DMEM/F12. The pellet was dissolved in 5 mL of DMEM/F12 and loaded on a pre-wetted 54- μm nylon filter to collect the mammary ductal fragments. To monodispense the ductal fragments, they were washed, trypsinized, and passed through a 40- μm cell strainer. Frank mammary carcinomas were dissected from female susceptible congenic control rats at 15 weeks after DMBA. To monodisperse frank mammary carcinomas, tissue was scissor-minced finely over ice, collagenase-treated, and trypsinized as described above.

After cell staining, 50,000 cells were acquired in a BD LSR II flow cytometer (BD Biosciences). The samples were compensated using single-stained cells using BD FACS-Diva software (BD Biosciences). T-cell subpopulations (CD4^+^, CD8^+^, γδTCR^+^, and CD161α) were gated and the mean fluorescence intensity (MFI) of CD62L was calculated by using FlowJo software (TreeStar Inc., Ashland, OR, USA). The CD62L^high ^gate was set such that the MFI of the CD62L^high^-expressing cells was fivefold the MFI of the total cells. For calculation of normalized MFI of CD62L, the MFI of different T-cell subpopulations in each experiment was normalized against the MFI of CD62L of CD4^+ ^T cells in susceptible congenic control animals from that experiment. Data were statistically analyzed by using Mann-Whitney non-parametric tests. Hoechst staining was used to gate live cells containing 2n-4n DNA.

### Cytokine expression measurement

Two million lymphocytes from spleen or inguinal mammary LNs were stimulated with 1 μg/mL concanavalin A (conA) (Calbiochem, now part of EMD Biosciences, Inc., San Diego, CA, USA) and were cultured in 2 mL of RPMI medium containing 10% fetal bovine serum for 24 hours at 37°C in a 95% air/5% CO_2 _atmosphere. Unstimulated cells served as a control. The expression levels of IL-2, IL-4, IL-6, IL-17, interferon-gamma (IFNγ), and tumor necrosis factor (TNF) cytokines in the supernatants were measured by enzyme-linked immunosorbent assay (ELISA). ELISA sets for rat IL-4, IL-6, IFNγ, and TNF were obtained from BD Biosciences. ELISA sets for rat IL-17 and IL-2 were purchased from eBioscience, Inc. (San Diego, CA, USA) and R&D Systems, Inc. (Minneapolis, MN, USA), respectively.

### *In vitro *T-cell proliferation

Splenic lymphocytes were stained with 1 μM carboxyfluorescein succinimidyl ester (CFSE) (Invitrogen Corporation) for 8 minutes at 37°C and washed three times using ice-cold RPMI medium containing 10% fetal calf serum. Two million lymphocytes were stimulated with 1 μg/mL conA and were cultured in 2 mL of RPMI medium containing 10% fetal bovine serum for 4 days at 37°C in a 95% air/5% CO_2 _atmosphere. Unstimulated cells served as a control. Cells were stained with fluorochrome-conjugated antibodies against rat CD3, CD4, CD8, γδTCR, or isotype controls and fixed in 1% formaldehyde. Cell proliferation was measured by dye dilution by using a BD LSR II flow cytometer. Percentages of cells that showed a decrease in CFSE fluorescence intensity were calculated by using FlowJo software and were defined as daughter cells in various T-cell subpopulations. In each experiment, the percentage of daughter cells in different T-cell subpopulations was normalized against the percentage of CD4^+ ^daughter T cells in the susceptible congenic control group from that experiment.

### CD25 and CD134 expression and estimation of intracellular reduced thiols and mitochondrial membrane potential

Unstimulated cells and activated cells were stained with fluorochrome-conjugated antibodies against rat CD3, CD25, and CD134 or isotype controls and fixed in 1% formaldehyde, and 20,000 cells were acquired in a BD LSR II flow cytometer. Percentages of CD3^+ ^T cells that expressed CD25 or CD134 were calculated by using FlowJo software. To measure intracellular reduced thiols, in cultured T cells, monochlorobimane (MCB) (final concentration of 40 μM, 30 minutes at 37°C) was loaded into cells. The cells were further stained with fluorochrome-conjugated rat CD3 antibody and were acquired on a flow cytometer. MCB fluorescence was measured at wavelengths of 350 nm (excitation) and 450 nm (emission). Mean MCB fluorescence in T cells was calculated by using FlowJo software. To measure mitochondrial membrane potential in cultured T cells, JC1 dye (final concentration of 2.5 μg/mL, 30 minutes at 37°C) was loaded into cells. The cells were further stained with fluorochrome-conjugated rat CD3 antibody and were acquired on a flow cytometer. JC1 was excited by using a 488-nm laser, and emission was measured at wavelengths of 530 and 575 nm. The ratio of yellow to green fluorescence in T cells was calculated by using FlowJo software.

## Results

### *Mcs5a *acts during early mammary cancer progression

**Figure 1:**
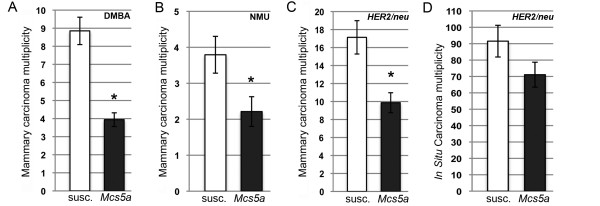
***Mcs5a *acts beyond mammary epithelial cell transformation during early cancer progression**. **(a) **7,12-Dimethylbenz(a)anthracene (DMBA)-induced mammary carcinoma multiplicities (susc., n = 27; *Mcs5a*, n = 49). **(b) ***N*-nitroso-*N*-methylurea (NMU)-induced mammary carcinoma multiplicities (susc., n = 29; *Mcs5a*, n = 28). **(c) **Mammary carcinoma multiplicities induced by mammary ductal infusion of retrovirus expressing the activated *HER2/neu *oncogene (*HER2/neu*) (susc., n = 15; *Mcs5a*, n = 15). **(d) ***HER2/neu*-induced *in situ *carcinoma multiplicities (susc., n = 24; *Mcs5a*, n = 24). Values are expressed as average ± standard error of the mean. Significance (*P *< 0.05) is indicated by an asterisk. *Mcs5a*, resistant congenic harboring the mammary carcinoma susceptibility locus 5a resistance allele; susc., susceptible congenic control.

The rat *Mcs5a *locus was fine-mapped as a DMBA-induced mammary carcinoma multiplicity phenotype. To rule out the possibility that *Mcs5a *affects the metabolic activation of DMBA, mammary carcinomas were induced by two additional treatments, namely carcinogenesis using the directly alkylating agent NMU and mammary ductal infusion of replication-defective retrovirus expressing the activated *HER2/neu *oncogene (*HER2/neu*) [15]. DMBA-, NMU-, and *HER2/neu*-induced mammary carcinoma multiplicities were compared between the susceptible congenic control line and the *Mcs5a*-resistant congenic line. Resistance to DMBA-, NMU-, and *HER2/neu*-induced mammary carcinogenesis was found to be 55%, 42%, and 42%, respectively (Figure 1a-c). *Mcs5a *conferred resistance to mammary carcinoma development induced by all three agents, suggesting that *Mcs5a *acts beyond the stage of cancer initiation. This was further investigated by assessing whether *Mcs5a *confers resistance to the formation of rapidly developing mammary carcinomas, termed ISCs [16]. Therefore, ISC multiplicity was determined 16 days after *HER2/neu *infusion. Susceptible congenic control and *Mcs5a*-resistant congenic rats did not differ in their average ISC multiplicity (Figure 1d). This result is compatible with a hypothesis that *Mcs5a *acts through a mechanism during early mammary cancer progression.

### *Mcs5a *acts through a non-mammary cell-autonomous mechanism

**Figure 2:**
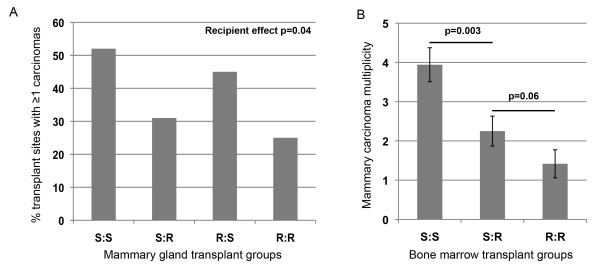
***Mcs5a *acts in a non-mammary cell-autonomous fashion through cells of the immune system**. **(a) **Percentage of transplant sites with one or more carcinomas in the mammary gland transplantation assay. Transplant groups are indicated as donor/recipient. Logistic regression analysis indicated a significant effect (*P *= 0.04) of the recipient on transplant site carcinoma incidence (Table 1). The animals in susceptible/susceptible (S:S), susceptible/resistant (S:R), resistant/susceptible (R:S), and resistant/resistant (R:R) transplant groups numbered 31, 72, 22, and 88, respectively. **(b) **7,12-Dimethylbenz(a)anthracene-induced mammary carcinoma multiplicities (expressed as average ± standard error of the mean) after bone marrow transplantation. Transplant groups are indicated as donor/recipient. Transplant group S:R (carrying an average immune system replacement of 53%; n = 16) displays a mammary carcinoma multiplicity intermediate to transplant groups R:R (n = 17) and S:S (n = 12). *Mcs5a*, mammary carcinoma susceptibility locus 5a.

To functionally investigate whether *Mcs5a *acts via a mammary cell-autonomous mechanism, a mammary gland transplantation assay was conducted. Mammary gland tissue from donor susceptible congenic control or *Mcs5a*-resistant congenic animals was transplanted into the interscapular white fat pads of recipient congenic animals of the same or opposite *Mcs5a *genotype. For the four transplant groups (S:S, S:R, R:S, and R:R), transplantation efficiency and carcinoma development following DMBA exposure were monitored (Table 1). There was no difference in the mammary tissue transplantation rate associated with donor and recipient or the interaction between donor and recipient genotypes (Table 1), ensuring that graft rejection was not a confounding variable in these congenic lines. The rates of mammary carcinoma incidence at the transplant site were 52%, 31%, 45%, and 25% for transplant groups S:S, S:R, R:S, and R:R, respectively (Figure 2a). Logistic regression analysis revealed that recipient genotype (*P *= 0.04), but not donor genotype (*P *= 0.65), was significantly associated with transplant site carcinoma development (Table 1). The interaction between donor and recipient genotype was not significant (*P *= 0.96) for the dependent variable mammary gland transplant carcinoma susceptibility (Table 1). These data demonstrate that the mammary carcinoma susceptibility phenotype mediated by *Mcs5a *is not transferable by transplantation of the mammary gland, indicating that *Mcs5a *does not act in a mammary cell-autonomous manner.

**Table 1:** Mammary gland transplantation data and logistic regression analysis

MG transplantation data^a^						Logistic regression analysis^b^			
	**S:S**	**S:R**	**R:S**	**R:R**	**Total**		**Independent**	**Coefficient**	***P *value**
Transplant MG present	31	72	22	88	213	MG transplantation efficiency	Donor effect	-0.3502	0.6449
Transplant MG absent	6	11	3	11	31		Recipient effect	-0.4372	0.4423
Percentage of MG present	84%	87%	88%	89%	87%		Donor × recipient	0.3389	0.7056
0 carcinomas	15	50	12	66	143				
1 carcinoma	13	17	10	19	59	MG transplant carcinoma susceptibility	Donor effect	-0.2468	0.6588
2 carcinomas	3	4	0	3	10		Recipient effect	-0.8855	0.0447^c ^
3 carcinomas	0	1	0	0	1		Donor × recipient	0.0307	0.9629
Percentage of MGs with 1 carcinoma	52%	31%	45%	25%	33%				

^a^Transplant groups (donor/recipient): S:S, susceptible/susceptible; S:R, susceptible/resistant; R:S, resistant/susceptible; R:R, resistant/resistant. ^b^Logistic regression was used to estimate the effect of donor and recipient and the interaction between donor and recipient for the dependent variables mammary gland (MG) transplantation efficiency (outcome = histologically determined presence or absence of MG development at transplant site) and MG transplant carcinoma susceptibility (outcome = tumor presence or absence at transplant site) in susceptible congenic control and *Mcs5a*-resistant congenic reciprocal (donor, recipient) transplant groups. ^c^Statistically significant.

### *Mcs5a *acts within immune cells to mediate mammary carcinoma susceptibility

The regulatory activity of the non-protein-coding *Mcs5a *locus on the expression of genes located within 1 Mb was found to manifest in the immune system and not in the mammary gland [5]. Therefore, we hypothesize that components of the immune system mediate the activity of *Mcs5a *in controlling the mammary carcinoma susceptibility phenotype. To evaluate this, a bone marrow transplantation assay was carried out. *Mcs5a*-resistant congenic and susceptible congenic control female recipients were irradiated to eliminate their bone marrow progenitor cells and grafted with bone marrow cells from either *Mcs5a*-resistant congenic or susceptible congenic control donors yielding four transplant groups (donor/recipient): S:S, S:R, R:S, and R:R. Graft levels of the immune system were quantified by using a TaqMan allelic discrimination assay on whole-spleen genomic DNA, with dilutions of genomic DNA of the respective homozygous *Mcs5a *genotypes as standards. At a dose of 2 × 400 rads, average replacement levels of greater than 50% were obtained in the S:R group in two out of four trials. Only data from the two experiments yielding greater than 50% of average replacement were included. Average replacement levels of greater than 50% were obtained in the R:S group in none of four trials and therefore could not be included in the analysis. A higher dose (2 × 500 rads) to get potentially higher levels of replacement resulted in cell killing in mammary gland and ovarian tissues at levels that preclude the development of mammary carcinomas for quantitative analysis (data not shown). At the dose of 2 × 400 rads, the average (± standard error of the mean) DMBA-induced mammary carcinoma multiplicity for the resistant control group R:R (1.4 ± 0.3) was reduced by approximately 64% as compared with the susceptible control group S:S (3.9 ± 0.4) (Figure 2b). The S:R transplant group yielded an intermediate average number of mammary carcinomas (2.2 ± 0.4). These data suggest that *Mcs5a *acts through components of the immune system to modulate mammary carcinoma susceptibility.

### *Mcs5a *modulates T-cell homeostasis and functions

**Figure 3:**
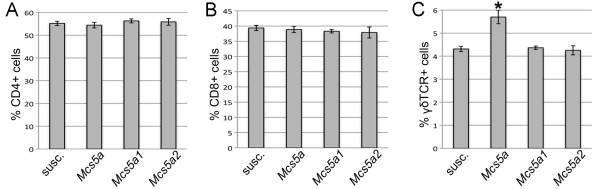
***Mcs5a *modulates γδTCR^+ ^T-cell homeostasis**. Percentages of **(a) **CD4^+^, **(b) **CD8^+^, and **(c) **γδTCR^+ ^T cells in the splenic CD3^+ ^T-cell population from susceptible congenic control (n = 26), *Mcs5a*-resistant congenic (n = 26), *Mcs5a1 *susceptible congenic (n = 20), and *Mcs5a2 *susceptible congenic (n = 15) rats. The significantly different (*P *< 0.05) γδTCR^+ ^T-cell population is indicated by an asterisk. Values are expressed as average ± standard error of the mean. *Mcs5a*, resistant congenic harboring the mammary carcinoma susceptibility locus 5a resistance allele; susc., susceptible congenic control; TCR, T-cell receptor.

We used the congenic rat lines to identify the target cell type in the immune system for the gene-regulatory activity of *Mcs5a*. The transcript level downregulation of *Fbxo10 *in thymocytes, primary T cells, cultured unstimulated T cells, and cultured conA-stimulated T cells appeared to be associated with the presence of the synthetically interacting genetic elements of the resistant *Mcs5a *allele [14]. Using immunophenotyping assays on T cells from spleen, inguinal mammary LN, and mammary epithelium from *Mcs5a*-resistant congenic (*Mcs5a*) and susceptible congenic rat lines (susc., *Mcs5a1 *and *Mcs5a2*), we investigated whether functional characteristics and phenotypes of various T-cell subpopulations are also associated with the *Mcs5a*-resistant allele. If a certain phenotype is present in the *Mcs5a*-resistant congenic animals only and not in the susceptible congenic animals (susc., *Mcs5a1 *and *Mcs5a2*), it is associated with the *Mcs5a*-resistant allele. The percentages of CD4^+ ^T cells and CD8^+ ^T cells in the CD3^+ ^T-cell population were found to be similar in spleens from resistant and susceptible congenic rat lines (Figure 3a, b). However, the proportion of γδTCR^+ ^T cells among the CD3^+ ^T cells was significantly higher (*P *< 0.0001) for *Mcs5a*-resistant congenic rats (Figure 3c).

**Figure 4:**
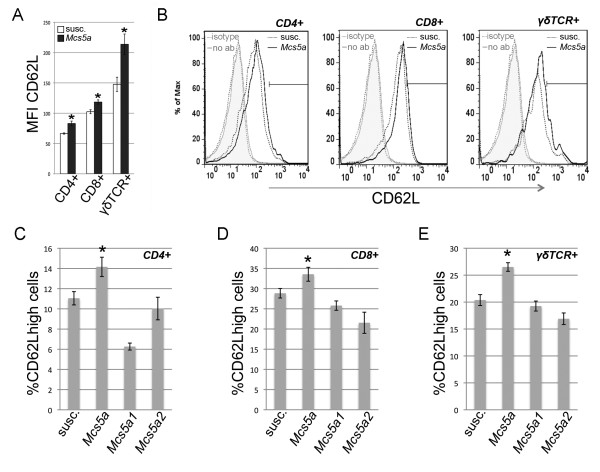
***Mcs5a *increases the CD62L^high ^population among CD4^+^, CD8^+^, and γδTCR^+ ^splenic T cells**. **(a) **CD62L mean fluorescence intensities (MFIs) of the splenic CD4^+^, CD8^+^, and γδTCR^+ ^T cells isolated from susceptible congenic control (open bars, n = 26) and *Mcs5a*-resistant congenic (filled bars, n = 29) rats. Significantly different (*P *< 0.05) CD62L expression is indicated by an asterisk. **(b) **Examples of overlaid flow cytometric histograms representing CD62L expression in CD4^+^, CD8^+^, and γδTCR^+ ^T cells in susceptible congenic control rats (dotted black line) and *Mcs5a*-resistant congenic rats (solid black line). The grey dotted line and grey solid line represent fluorescence in the CD62L channel of an isotype control antibody-stained (isotype) and unstained (no ab) cell sample, respectively. The black bar represents the gate used to quantify the CD62L^high ^population in each T-cell subpopulation; the gate was set such that the MFI of the high population was fivefold that of the total cells. **(c) **Percentage of CD62L^high^-gated cells among CD4^+ ^splenic T cells. **(d) **Percentage of CD62L^high^-gated cells among CD8^+ ^splenic T cells. **(e) **Percentage of CD62L^high^-gated cells among γδTCR^+ ^splenic T cells. CD62L^high^-expressing T cells of susceptible congenic control (susc., n = 26), *Mcs5a*-resistant congenic (*Mcs5a*, n = 26), *Mcs5a1 *susceptible congenic (*Mcs5a1*, n = 20), and *Mcs5a2 *susceptible congenic (*Mcs5a2*, n = 15) rat lines are shown. A significantly higher (*P *< 0.05) percentage of CD62L^high ^cells in the *Mcs5a*-resistant congenic line compared with the three susceptible congenic lines is indicated by an asterisk. Values in (a, c-e) are expressed as average ± standard error of the mean. *Mcs5a*, mammary carcinoma susceptibility locus 5a; TCR, T-cell receptor.

**Figure 5:**
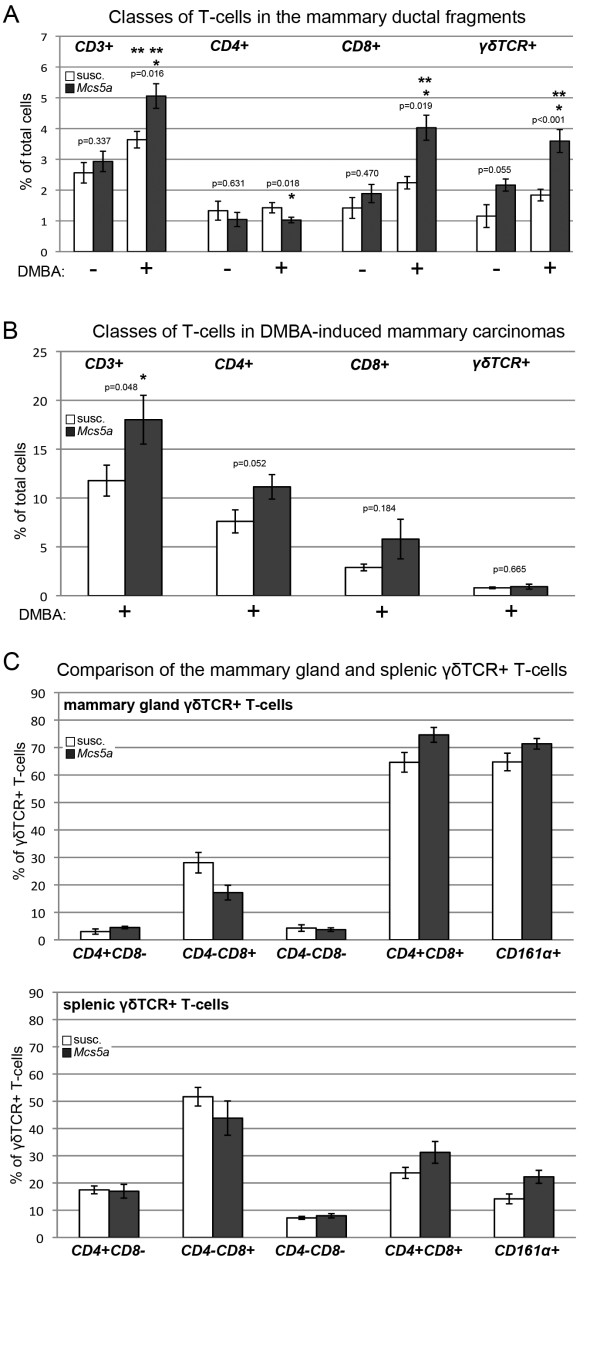
***Mcs5a *is associated with an increased CD3^+ ^T-cell abundance attributable to increased γδTCR**^+ ^**T-cell abundance in the mammary epithelium of DMBA-treated rats as compared with untreated rats**. **(a) **Percentage of CD3^+^, CD4^+^, CD8^+^, or γδTCR^+ ^T cells of total cells. Values are expressed as average ± standard error of the mean. Significantly higher (*P *< 0.05) percentages in the *Mcs5a*-resistant congenic rat line (*Mcs5a*, filled bars; -DMBA n = 6, +DMBA n = 12) compared with the susceptible congenic control line (susc., open bars; -DMBA n = 6, +DMBA n = 12) are indicated by an asterisk. Significantly higher (*P *< 0.05) percentage of T-cell abundance upon DMBA treatment compared with no treatment is indicated by two asterisks. **(b) **Infiltrating T cells in frank mammary carcinomas of susceptible (open bars, n = 11) and *Mcs5a*-resistant congenic rat line (filled bars, n = 12) rats. Significantly higher (*P *< 0.05) percentages in the *Mcs5a*-resistant congenic rat line compared with the susceptible congenic control line are indicated by an asterisk. **(c) **Expression of CD4, CD8, and CD161α on γδTCR^+ ^T cells in mammary gland and spleen of susceptible (open bars, n = 6) and *Mcs5a*-resistant congenic rat line (filled bars, n = 7) rats. DMBA, 7,12-dimethylbenz(a)anthracene; *Mcs5a*, mammary carcinoma susceptibility locus 5a; TCR, T-cell receptor.

The expression of L-selectin (CD62L) was significantly higher on CD4^+ ^(*P *= 0.0001), CD8^+ ^(*P *= 0.0036), and γδTCR^+ ^(*P *= 0.0023) T cells from the spleen of the *Mcs5a*-resistant congenic rats (Figure 4a, b). This observation can be attributed to higher levels of CD62L^high^-expressing cells in all T-cell subpopulations from *Mcs5a*-resistant congenic rats compared with the susceptible congenic rat lines (Figure 4c-e). The role of L-selectin in the homing of T cells to secondary lymphoid organs and sites of inflammation in extralymphoid organs has been well established [17]. Therefore, the composition of the T-cell compartment of the mammary epithelium of untreated and DMBA-treated rats was examined. The mammary epithelium of untreated *Mcs5a*-resistant congenic rats as compared with susceptible congenic control rats had equal percentages of CD3^+ ^T cells (Figure 5a), of which CD4^+ ^and CD8^+ ^T cells were equal as well, but γδTCR^+ ^T cells showed a strong trend (*P *= 0.055) toward overabundance (Figure 5a). After DMBA treatment, CD3^+ ^T-cell abundance in the mammary epithelium increased in both susceptible congenic control (*P *= 0.031) and *Mcs5a*-resistant congenic (*P *= 0.007) rats as compared with the untreated mammary epithelium (Figure 5a). This increase is attributable to the CD8^+ ^or γδTCR^+ ^T cells or both and not attributable to the CD4^+ ^T cells. Subsequently, DMBA-treated *Mcs5a*-resistant congenic rats were found to have significantly higher percentages of CD3^+ ^(*P *= 0.016), CD8^+ ^(*P *= 0.018), and/or γδTCR^+ ^(*P *< 0.001) T cells and a significantly lower percentage of CD4^+ ^T cells (*P *= 0.019) as compared with susceptible congenic control rats (Figure 5a). Next, we examined the same classes of T cells within DMBA-induced mammary carcinomas (Figure 5b). We found an overabundance (*P *= 0.048) of CD3^+ ^T cells in the mammary carcinomas from the *Mcs5a*-resistant congenic rats compared with the susceptible congenic control rats. Interestingly, these CD3^+ ^T cells consisted mainly of CD4^+ ^and CD8^+ ^αβTCR^+ ^T cells, and γδTCR^+ ^T cells were a minor population (Figure 5b). The mammary carcinoma T cells do not appear to be activated, since they did not produce IFNγ (data not shown).

As a result of the observation that γδTCR^+ ^T cells in the normal mammary epithelium seem to also express CD8 (Figure 5a), we characterized the mammary gland γδTCR^+ ^T-cell population in comparison with the splenic γδTCR^+ ^T-cell population (Figure 5c). In contrast to splenic γδTCR^+ ^T cells, most of the mammary gland γδTCR^+ ^T cells expressed the CD4, CD8, and CD161α markers simultaneously (Figure 5c).

**Figure 6:**
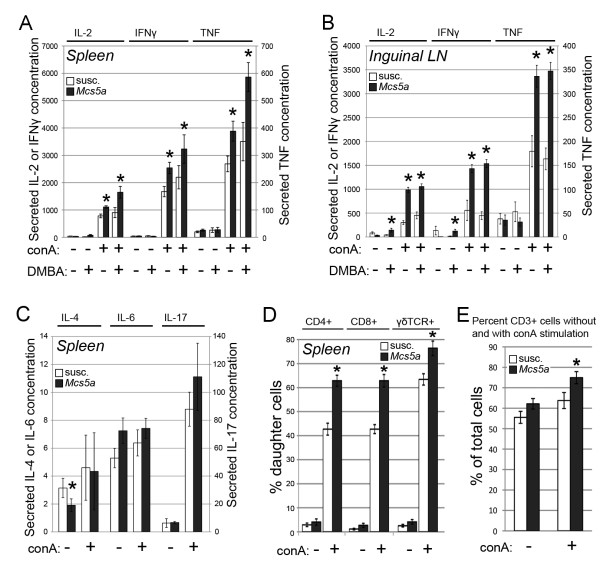
***Mcs5a *allele is associated with increased concanavalin A (conA)-induced Th1 cytokine production and proliferation**. **(a) **Interleukin-2 (IL-2), interferon-gamma (IFNγ), or tumor necrosis factor (TNF) concentrations (in picograms per milliliter) in the media produced by cultured T cells from spleens of susceptible congenic control (susc., open bars; -DMBA n = 30, +DMBA n = 8) and *Mcs5a *congenic resistant (*Mcs5a*, filled bars; -DMBA n = 30, +DMBA n = 8) rats without or with conA stimulation. **(b) **IL-2, IFNγ, or TNF concentrations (in picograms per milliliter) in the media produced by cultured T cells from inguinal mammary lymph nodes (LNs) of susceptible congenic control (open bars; -DMBA n = 8, +DMBA n = 8) and *Mcs5a *congenic resistant (filled bars; -DMBA n = 8, +DMBA n = 8) rats without or with conA stimulation. **(c) **IL-4, IL-6, or IL-17 concentrations (in picograms per milliliter) in the media produced by cultured T cells from spleens of susceptible congenic control (open bars, n = 22) and *Mcs5a *congenic resistant (filled bars, n = 22) rats without or with conA stimulation. **(d) **Percentage of second- and later-generation daughter cells among CD4^+^, CD8^+^, and γδTCR^+ ^T cells from susceptible congenic control (open bars, n = 24) and *Mcs5a*-resistant congenic (filled bars, n = 26) rats without or with conA stimulation. Significantly different (*P *< 0.05) cytokine production or proliferation between susceptible congenic control and *Mcs5a*-resistant congenic rat lines is indicated by an asterisk. Values are expressed as average ± standard error of the mean. DMBA, 7,12-dimethylbenz(a)anthracene; *Mcs5a*, mammary carcinoma susceptibility locus 5a; TCR, T-cell receptor.

To evaluate whether *Mcs5a *affects T-cell functions, cytokine and proliferation assays were carried out. ConA-activated splenic T cells from *Mcs5a*-resistant congenic rats produced significantly higher levels of Th1 cytokines IL-2 (*P *= 0.0001), IFNγ (*P *= 0.0033), and TNF (*P *= 0.0045) as compared with T cells from susceptible congenic control rats (Figure 6a). These differences were found to be more pronounced when analyzing the conA-induced Th1 cytokine production of T cells from inguinal mammary LNs (Figure 6b). The differential production of Th1 cytokines by susceptible congenic control and *Mcs5a*-resistant congenic rats was maintained after DMBA treatment (Figure 6a, b). The conA-induced production of Th2 cytokines IL-4 (*P *= 0.61) and IL-6 (*P *= 0.27) and the Th17 cytokine IL-17 (*P *= 0.16) by splenic T cells was not significantly different (Figure 6c). Interestingly, mitogen (conA)-induced proliferation of different splenic T-cell subpopulations was also significantly different in *Mcs5a*-resistant congenic rats as compared with susceptible congenic control rats. The percentage of daughter cells that had undergone more than two divisions was significantly higher in CD4^+ ^(*P *< 0.0001), CD8^+ ^(*P *< 0.0001), and γδTCR^+ ^(*P *= 0.0009) T cells from *Mcs5a*-resistant congenic rats as compared with the susceptible congenic control rats (Figure 6d). As a result, the percentage of total T cells at the end of the proliferation experiment was increased in the *Mcs5a*-resistant congenic samples (Figure 6e).

We looked at the expression of markers CD25 and CD134 in the T cells without or with conA stimulation. The expression of CD25 was lower (*P *= 0.02) in the *Mcs5a*-resistant congenic animals after conA stimulation (Figure S1A of Additional file 1). The expression of CD134 was not different between susceptible and *Mcs5a*-resistant congenic animals (Figure S1B of Additional file 1). Finally, we looked at the reduced thiol levels and mitochondrial membrane potential in the T cells without or with conA stimulation. Both of these parameters were significantly higher in the *Mcs5a*-resistant congenic animals (*P *= 0.007 and *P *< 0.001) after conA stimulation.

## Discussion

*MCS5A/Mcs5a *is a non-protein-coding locus that associates with breast cancer risk in women and rats. Here, we have shown that the resistant rat *Mcs5a *allele prevents cancer induced by three distinctly acting mammary carcinoma-inducing treatments, indicating that *Mcs5a *does not control a specific initial step of mammary epithelial cell transformation. In addition, formation of ISCs [15] was not affected by *Mcs5a*, suggesting that *Mcs5a *acts during early carcinoma progression. In the mammary gland transplantation assay, it was demonstrated that *Mcs5a *acts in a non-mammary cell-autonomous fashion. This, together with the observation that *Fbxo10 *differential expression manifests only in T cells [14], led to the hypothesis that *Mcs5a *does not solely function in the mammary parenchyma to modulate mammary cancer susceptibility but instead acts through the immune system. To assess the involvement of the immune system, we conducted a bone marrow transplantation assay, in which we used irradiation to eliminate the recipient rat bone marrow in order to replace it with bone marrow cells from donor rats of the same or opposite *Mcs5a *genotype. At the maximal dose of radiation, only partial replacement could be achieved as a higher dose of radiation resulted in ovarian and mammary organ damage that compromised mammary carcinoma development. At the maximal dose of radiation, the mammary carcinoma multiplicities of the control transplant groups S:S and R:R were approximately twofold lower compared with the average DMBA-induced mammary carcinoma multiplicity routinely obtained in non-irradiated rats of the same genotypes (Figure 1a) and this was due to radiation organ damage. Nevertheless, partial replacement (average of greater than 50%) of the immune cells of the *Mcs5a*-resistant congenic rats with immune cells of the susceptible congenic control rats (transplant group S:R) yielded a mammary carcinoma multiplicity intermediate to the control transplant groups R:R and S:S. This intermediate phenotype of partially reconstituted rats is quantitatively comparable to the intermediate mammary carcinoma multiplicity phenotype of rats that are genetically heterozygous for the *Mcs5a*-resistant allele [5]. We concluded that *Mcs5a *acts through components of the immune system to modulate mammary carcinoma multiplicity.

Specific T-cell phenotypes are also under control of the synthetically interacting *Mcs5a *elements that control *Fbxo10 *transcript levels and mammary carcinoma multiplicity. We found an overabundance of γδTCR^+ ^T cells (important in mucosal cell surface surveillance), but not CD4^+ ^(T helper) and CD8^+ ^(cytotoxic) T cells, in the spleen of *Mcs5a*-resistant congenic rats compared with susceptible congenic rats. In addition, the CD4^+^, CD8^+^, and γδTCR^+ ^T-cell populations harbored an increased percentage of CD62L^high^-expressing cells in *Mcs5a*-resistant congenic rats. The resistant *Mcs5a *allele was also found to be associated with an increased proliferation rate of activated splenic T cells as well as the production of the Th1 cytokines IL-2, IFNγ, and TNF of activated T cells from spleen and, to a greater extent, from inguinal mammary LNs. The production of Th2 or Th17 cytokines was not affected by *Mcs5a*. T cells from *Mcs5a*-resistant congenic rats showed lower CD25 expression (IL2Rα) but higher cellular reduced thiol levels and mitochondrial membrane potential and increased IL-2 production. Considering these results, we propose that higher proliferative response in *Mcs5a*-resistant congenic T cells could be due primarily to increased cytokine signaling and lower oxidative stress.

Considering the altered T-cell homeostasis and functionality associated with the resistant *Mcs5a *allele, we speculate that T cells act at the mammary gland to control mammary carcinoma susceptibility. To begin to explore this hypothesis, we compared the T-cell population within the mammary epithelium between the susceptible congenic control and the *Mcs5a*-resistant congenic rats. We found a higher abundance of mammary epithelium-residing CD3^+ ^T cells, mainly consisting of γδTCR^+ ^T cells in the DMBA-treated *Mcs5a*-resistant congenic rats. In contrast to the splenic γδTCR^+ ^T cells, most of the γδTCR^+ ^T cells in the mammary gland are double-positive for CD4 and CD8 and also expressed NK-receptor P1 CD161α. This result suggests that the mammary γδTCR^+ ^T cells may harbor increased cytotoxic characteristics that are potentially capable of killing emerging tumor cells. In contrast to normal and carcinogen-exposed mammary gland, frank mammary carcinomas have a low abundance of γδTCR^+ ^T cells. The mammary carcinoma T-cell population consists mainly of αβTCR^+ ^T cells. This raises an interesting possibility that immunoprevention of breast cancer versus breast cancer therapy may need to focus on different T-cell class targets, namely γδTCR^+ ^T cells for prevention and αβTCR^+ ^T cells for therapy.

Increased γδTCR^+ ^T-cell abundance in the mammary epithelium is consistent with the observations that *Mcs5a*-resistant congenic rats have an increased population of CD62L^high^-expressing T cells and that CD62L (L-selectin) expression was highest in γδTCR^+ ^as compared with CD4^+ ^and CD8^+ ^splenic T cells (Figure 4a). L-selectin is a cell adhesion protein involved in leukocyte-endothelial 'rolling' to facilitate extravasation of leukocytes from blood and lymphatic vessels and in homing of leukocytes to secondary LNs and tumors in extralymphoid organs [17-19].

Interestingly, γδTCR^+ ^T cells have been implicated in the protection against breast cancer in women. Decreased peripheral abundance of γδTCR^+ ^T cells and their diminished IFNγ production were recently shown to be associated with breast cancer [20]. Treatment of osteoporosis with bisphosphonates, compounds known as γδTCR^+ ^T-cell agonists [21], has been associated with reduced breast cancer risk [22]. Additionally, the Th1 cytokine IFNγ was implicated in tumor immune surveillance in mice [23].

Here, we have shown that the human-rat conserved non-protein-coding *MCS5A/Mcs5a *locus acts in a non-mammary cell-autonomous fashion through the immune system and modifies the homeostasis and functions of T cells. Each of these phenotypes or a combination of them has the potential to underlie the mammary carcinoma resistance phenotype mediated by the resistant *Mcs5a *allele. Possible mechanisms include immune surveillance or immune cell-produced Th1 cytokines altering the cellular composition/differentiation of the mammary parenchyma or both. In the future, additional mechanistic studies on the immunological aspect of *Mcs5a *will address the contribution of each of the observed T-cell phenotypes to *Mcs5a*-mediated mammary carcinoma resistance.

## Conclusions

The non-protein-coding human-rat conserved mammary carcinoma susceptibility locus *Mcs5a *modulates mammary tumor multiplicity through a mechanism beyond the initial stage of epithelial cell transformation, during early cancer progression. *Mcs5a *acts in a non-mammary cell-autonomous manner through the immune system. The resistant allele is associated with an overabundance of γδTCR^+ ^T cells in the spleen, an overabundance of CD62L^high^-expressing cells of all T-cell classes, and, importantly, an overabundance of γδTCR^+ ^T cells (CD4^+^, CD8^+^, and CD161α^+^) in the mammary epithelium after treatment with the carcinogen DMBA. γδTCR^+ ^T cells are the most abundant T-cell class in the mammary epithelium but not in mammary carcinomas. The resistant *Mcs5a *allele is also associated with increased proliferation and Th1, but not Th2 or Th17, cytokine secretion of activated T cells. We hypothesize that the resistant *Mcs5a *allele acts through specific cells of the immune system to prevent mammary carcinoma development.

## Abbreviations

CFSE: carboxyfluorescein succinimidyl ester; CFU: colony-forming unit; ConA: concanavalin A; DMBA: 7,12-dimethylbenz(a)anthracene; DMEM/F12: Dulbecco's modified Eagle's medium/F12; ELISA: enzyme-linked immunosorbent assay; IFNγ: interferon-gamma; IL: interleukin; ISC: *in situ *carcinoma; LN: lymph node; MCB: monochlorobimane; *Mcs5a*: mammary carcinoma susceptibility locus 5a; MFI: mean fluorescence intensity; NMU: *N*-nitroso-*N*-methylurea; R: resistant; S: susceptible; susc.: susceptible congenic control; TCR: T-cell receptor; TNF: tumor necrosis factor; WF: Wistar-Furth; WKy: Wistar-Kyoto.

## Competing interests

The authors declare that they have no competing interests.

## Authors' contributions

BMGS, DS, and DJS performed experiments, analyzed data, and drafted the manuscript. SW and JDH performed experiments and analyzed data. BM designed statistical approaches and analyzed data. MNG conceived the study, participated in the design of the study, and helped to draft the manuscript. All authors read and approved the final manuscript.

## Supplementary Material

Additional file 1 ***Mcs5a *and T-cell associations**. *Mcs5a *is associated with decreased conA-induced CD25 upregulation in T-cells (Figure S1). *Mcs5a *is associated with activation induced changes in reduced thiol levels and mitochondrial membrane potential T-cells (Figure S2). Click here for file
