# Supplementary material for: The non-protein coding breast cancer susceptibility locus Mcs5a acts in a non-mammary cell-autonomous fashion through the immune system and modulates T-cell homeostasis and functions
Source: Breast Cancer Res. 2011 Aug 16;13(4):R81. doi: 10.1186/bcr2933 (PMC3236344; doi:10.1186/bcr2933)
Supplement: Additional file 1 — Mcs5a and T-cell associations. Mcs5a is associated with decreased conA-induced CD25 upregulation in T-cells (Figure S1). Mcs5a is associated with activation induced changes in reduced thiol levels and mitochondrial membrane potential T-cells (Figure S2). [file bcr2933-S1.PDF]

ADDITIONAL FILE

Figure S1

A Percent T-cells expressing CD25 without and with conA-stimulation

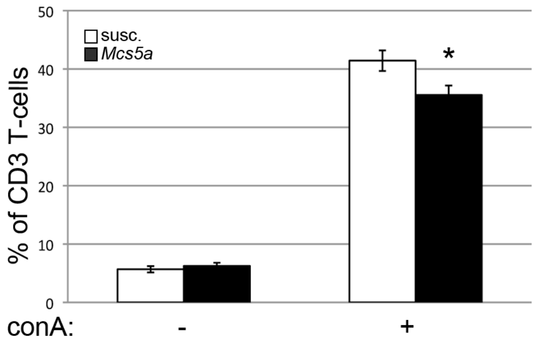

B Percent T-cells expressing CD134 without and with conA-stimulation

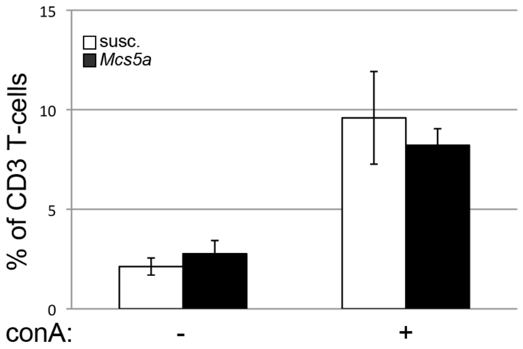

Figure S2

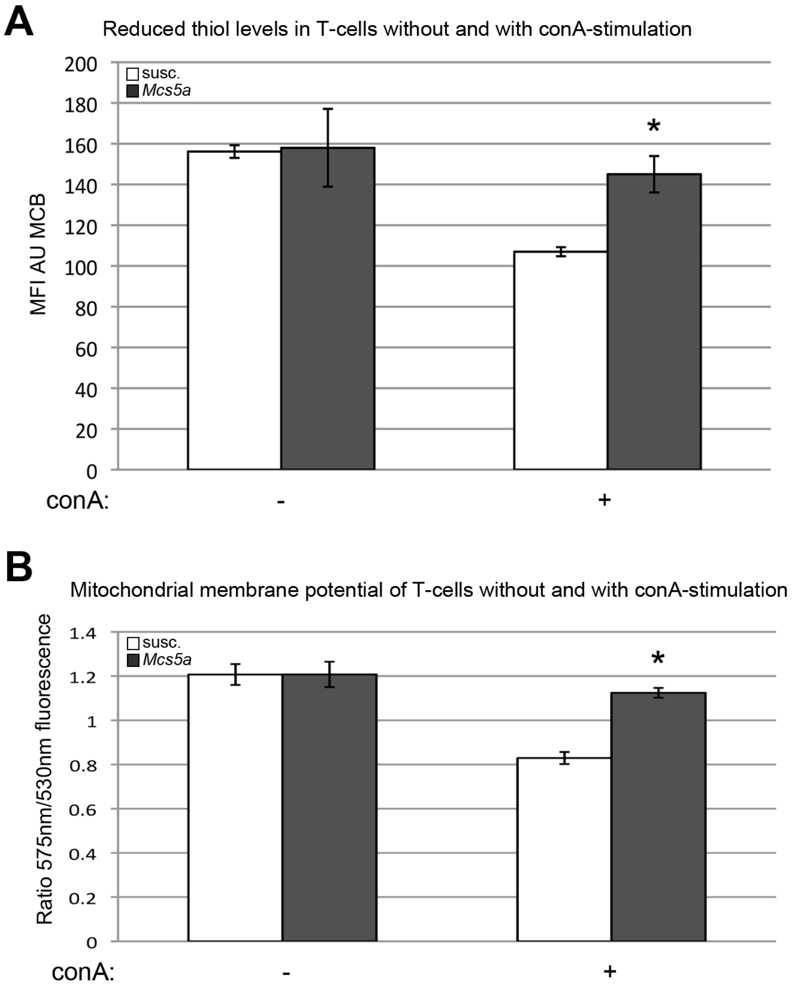

A
Reduced thiol levels in T-cells without and with conA-stimulation
MFI AU MCB
200
180
160
140
120
100
80
60
40
20
0
susc.
Mcs5a
*
conA:
-
+
B
Mitochondrial membrane potential of T-cells without and with conA-stimulation
Ratio 575nm/530nm fluorescence
1.4
1.2
1
0.8
0.6
0.4
0.2
0
susc.
Mcs5a
*
conA:
-
+

Figure S1: *Mcs5a* is associated with decreased conA-induced CD25 upregulation in T-cells. A. Average ± sem percentage of CD25+ T-cells from spleens of susceptible congenic control (open bars; n=22) and *Mcs5a* congenic resistant rats (filled bars; n=22) 24h after conA-stimulation. **B.** Average ± sem percentage of CD134+ T-cells from spleens of susceptible congenic control (open bars, n=7) and *Mcs5a* congenic resistant rats (filled bars; n=7) 24h after conA-stimulation. Significantly different (P<0.05) expression of CD25 on T-cells between susceptible congenic control and *Mcs5a* resistant congenic rat lines is indicated with an asterisk.

Figure S2: *Mcs5a* is associated with activation induced changes in reduced thiol levels and mitochondrial membrane potential T-cells. A. Average ± sem percentage of monochlorobimane (MCB) fluorescence intensity in T-cells from susceptible congenic control (open bars; n=6) and *Mcs5a* congenic resistant rats (filled bars; n=6) 24h after conA-stimulation. **B.** Average ± sem ratio of fluorescence emission at 575nm and 530nm wavelengths in JC1 stained T-cells from spleens of susceptible congenic control (open bars, n=7) and *Mcs5a* congenic resistant rats (filled bars; n=7) 24h after conA-stimulation. Significantly different (P<0.05) reduced thiol levels and mitochondrial membrane potential between susceptible congenic control and *Mcs5a* resistant congenic rat lines is indicated with an asterisk.
